# Bacterial Persistence Modulates the Speed, Magnitude, and Onset of Antibiotic Resistance Evolution

**DOI:** 10.1093/molbev/msag180

**Published:** 2026-07-22

**Authors:** Giorgio Boccarella, Philip Ruelens, Ernesto Berríos-Caro, Bram Van den Bergh, Lloyd Cool, Jan Michiels, Pieter van den Berg

**Affiliations:** Evolutionary Modelling Group, Department of Biology, KU Leuven, Leuven, Belgium; Centre of Microbial and Plant Genetics, KU Leuven, Leuven, Belgium; Center for Microbiology, VIB-KU Leuven, Leuven, Belgium; Centre of Microbial and Plant Genetics, KU Leuven, Leuven, Belgium; Center for Microbiology, VIB-KU Leuven, Leuven, Belgium; Research Group Stochastic Evolutionary Dynamics, Max Planck Institute for Evolutionary Biology, Plön, Germany; Center for Advanced Biotechnology and Medicine, Rutgers University, Piscataway, NJ, USA; Centre of Microbial and Plant Genetics, KU Leuven, Leuven, Belgium; Center for Microbiology, VIB-KU Leuven, Leuven, Belgium; Centre of Microbial and Plant Genetics, KU Leuven, Leuven, Belgium; Center for Microbiology, VIB-KU Leuven, Leuven, Belgium; Laboratory of Socioecology and Social Evolution, KU Leuven, Leuven, Belgium; Centre of Microbial and Plant Genetics, KU Leuven, Leuven, Belgium; Center for Microbiology, VIB-KU Leuven, Leuven, Belgium; Evolutionary Modelling Group, Department of Biology, KU Leuven, Leuven, Belgium; Centre of Microbial and Plant Genetics, KU Leuven, Leuven, Belgium

**Keywords:** antibiotic resistance, bacterial persistence, evolutionary model

## Abstract

Bacterial persistence, a phenomenon where a subpopulation of cells can exhibit increased survival to antibiotic stress, allows part of the population to withstand antibiotic treatment. This can have far-reaching implications for the evolution of antibiotic resistance, yet its evolutionary consequences remain insufficiently understood. Here, using a combination of evolutionary modeling and experimental evolution data on *Escherichia coli*, we show that persistence impacts the speed, onset, and magnitude of antibiotic resistance evolution. Specifically, high persistence facilitates the slow establishment of small-effect mutations by protecting them from antibiotic killing, while low persistence boosts the rapid yet unpredictable emergence of rare large-effect resistance mutations by amplifying their competitive advantage. Our results show that persistence level has a critical impact on the trade-off between short-term population survival and the evolution of high-level resistance, with distinct outcomes depending on the severity of the antibiotic treatment regimen. These findings provide key insights for designing antibiotic treatment strategies that minimize the risk of antibiotic resistance evolution while maintaining treatment efficacy.

## Introduction

The advent of antibiotics revolutionized medicine, enabling the effective treatment of previously fatal bacterial infections. However, the rapid emergence and spread of antibiotic resistance among bacterial populations now threaten to undermine these therapeutic gains, posing a significant global public health crisis (Willyard 2017; Naghavi et al. 2024). Designing effective strategies to combat antibiotic resistance requires a deeper understanding of the evolutionary mechanisms that drive its emergence (Andersson et al. 2020; Baquero et al. 2021).

Recent evidence shows that the evolution of antibiotic resistance can be facilitated by other bacterial strategies that allow survival in adverse conditions (Cohen et al. 2013; Dewachter et al. 2019; Merker et al. 2020). This is the case for bacterial persistence (Balaban et al. 2019; Brauner and Balaban 2021), where a subpopulation of cells transiently survives antibiotic stress without being genotypically different. Persistence in itself is also an important cause of treatment failure, but its mechanistic and evolutionary causes are still poorly understood (Verstraete et al. 2022; Bollen et al. 2023; Van den Bergh et al. 2025), obscuring its role in resistance evolution. Levin-Reisman and colleagues in their seminal publication showed that tolerance often evolves before resistance and that tolerance also facilitates resistance evolution by protecting partial resistance mutations from extinction (Levin-Reisman et al. 2017; Levin-Reisman et al. 2019). This is an important insight, but it does not give a full picture. The fitness advantage conferred by a resistance mutation depends jointly on the magnitude of resistance it provides and the severity of the antibiotic treatment. Under some conditions, persistence may shield nascent resistance mutations from antibiotic killing; under others, it may hinder their establishment by the increased competition from surviving wild-type cells. Consequently, it remains unclear how persistence impacts which resistance mutations are likely to emerge, and how their dynamics depend on the antibiotic treatment regimen.

In this study, we present an evolutionary model to systematically examine how persistence influences the evolution of resistance, and then we evaluate its predictions using experimental evolution data from *Escherichia coli*. We focus specifically on how persistence impacts the fate of resistance mutations of different magnitudes across antibiotic treatment regimens of varying severity. We find that persistence modulates a fundamental trade-off between survival and resistance evolution: while high-persistence populations tend to foster the gradual establishment of relatively common, small-effect resistance mutations, resulting in slow but predictable resistance evolution, low-persistence populations promote the rapid emergence of rarer large-effect mutations, leading to a faster but less predictable onset of resistance, at a greater risk of population extinction.

## Results

### Persistence affects the emergence of antibiotic resistance mutations

We start out with a simple mathematical model to capture how persistence level impacts the establishment of mutations that confer different levels of resistance, under a simple scenario of antibiotic exposure. Specifically, we model a population that is temporarily exposed to a constant concentration of antibiotics, followed by a regrowth window, in turn followed by a subsequent bout of antibiotic exposure. Our goal is to quantify the probability that at least one resistant mutant of the specified resistance level arises during the regrowth window and then also survives the second phase of antibiotic treatment.

We model survival during antibiotic exposure as determined by resistance minimum inhibitory concentration (MIC) and persistence. Survival is described by a standard pharmacodynamic function of antibiotic concentration with persistence modeled as a subpopulation-level increase in survival during antibiotic exposure driven by a lower antibiotic-induced killing rate. We specifically focus on this treatment-survival aspect of persistence, in line with the definition of persistence proposed by Balaban et al. (2019) (see Materials and methods), while abstracting away from the diverse physiological mechanisms (reduced metabolism, altered lag time, stress responses, delayed recovery) that characterize persister cells. Resistance mutations may occur during cell division in the regrowth phase, with the mutation rate $\mu_{r}$ depending on the resistance level that the mutation confers. We assume mutation rates follow a log-normal distribution, with small-effect mutations much more common than mutations that confer high resistance (see Materials and methods for details). To calculate the overall probability $P_{e,r}$ (“emergence probability”) that at least one mutant of resistance level *r* both arises during regrowth and survives the subsequent bout of antibiotic exposure, we integrate over all possible times of appearance *t*:


(1) $$P_{e,r}=1-\exp\;\left[-\int_{0}^{T}\;b_{\mathrm{wt}\;}\mu_{r}n_{\mathrm{wt}}(t)\;p_{\mathrm{est}}(t,T)S_{m,r}\;dt\right]$$

where $\;b_{\mathrm{wt}}$ is the birth rate of the wild-type population, $n_{\mathrm{wt}}(t)$ is the number of wild-type cells at time *t*, $p_{\mathrm{est}}(t,T)$ is the probability that a mutant arising at time *t* establishes a lineage that avoids stochastic loss by the end of the regrowth window (time *T*), and $S_{m,r}$ is the probability that the mutation survives the second bout of antibiotic treatment. The full derivation of this equation is presented in the SI Appendix.

**Figure 1:**
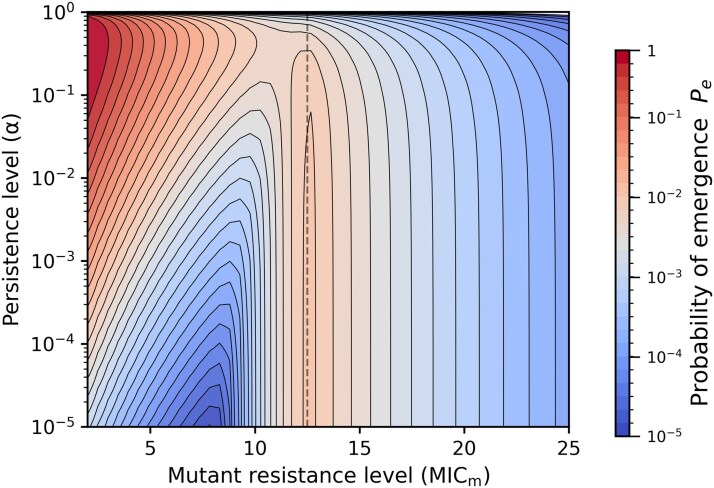
The emergence probability of resistance mutations, depending on the persistence level and the degree of resistance that the mutation confers. Colors show the predicted emergence probability (*P_e_*) of a mutation depending on the magnitude of the resistance mutation (*x* axis) and on the persistence level (*y* axis). Note that *α* = 1 corresponds not to a persister fraction but the limiting case of population-wide tolerance. In this example, the antibiotic concentration of the treatment is equal to 12.5 μg/ml (indicated by the vertical dashed line), and the wild-type MIC is 2 μg/ml.

Figure 1 shows how persistence impacts the emergence probability of resistance mutations of different magnitudes. While high persistence mainly facilitates the emergence of mutations conferring only modest MIC increases, low persistence mostly leads to the emergence of resistance mutations that confer relatively high resistance. This pattern occurs because emergence depends both on the likelihood that mutations arise and on the likelihood that they then survive demographic stochasticity and antibiotic exposure. The persistence level affects these two aspects in opposite directions: High persistence increases the survival of especially small-effect mutants, but it also shortens the regrowth window *T* (as regrowth starts from a larger surviving population), which limits opportunities for mutants to occur and expand. This favors the emergence of common, small-effect mutations, while disfavoring rare, large-effect mutations. Conversely, low persistence lengthens *T*, providing more opportunities for rare large-effect resistance mutants to arise and expand, and these mutants can subsequently survive the antibiotic exposure without needing protection from persistence. At the same time, small-effect resistance mutations rarely survive antibiotic exposure in this low-persistence scenario.

### Persistence affects the speed and predictability of the onset of resistance evolution

While the mathematical model illustrated in Fig. 1 provides insights into the establishment of single mutations in a wild-type background, it does not capture how resistance levels evolve over time when multiple mutations can arise and compete. To investigate this, we developed a simulation model based on the same core assumptions, now allowing both clonal interference—where multiple resistant lineages can arise and compete in parallel—and the incremental acquisition of resistance through multiple sequential mutations with a linear additive effect. We did this through implementing a variant of the Gillespie algorithm that tracks discrete lineages, each defined by its MIC and persistence phenotype (see Materials and methods). Key simulation parameters were chosen to align with the conditions of the experimental data discussed below. We simulated 12 consecutive 24 h cycles, where each cycle contains a 5 h antibiotic exposure period, during which a set fraction of the population transitions to the persistence state, followed by a 19 h regrowth period. Resistance-conferring mutations are again drawn from a continuous log-normal distribution and can occur at any birth event, yielding a new lineage with distinct MIC values.

**Figure 2:**
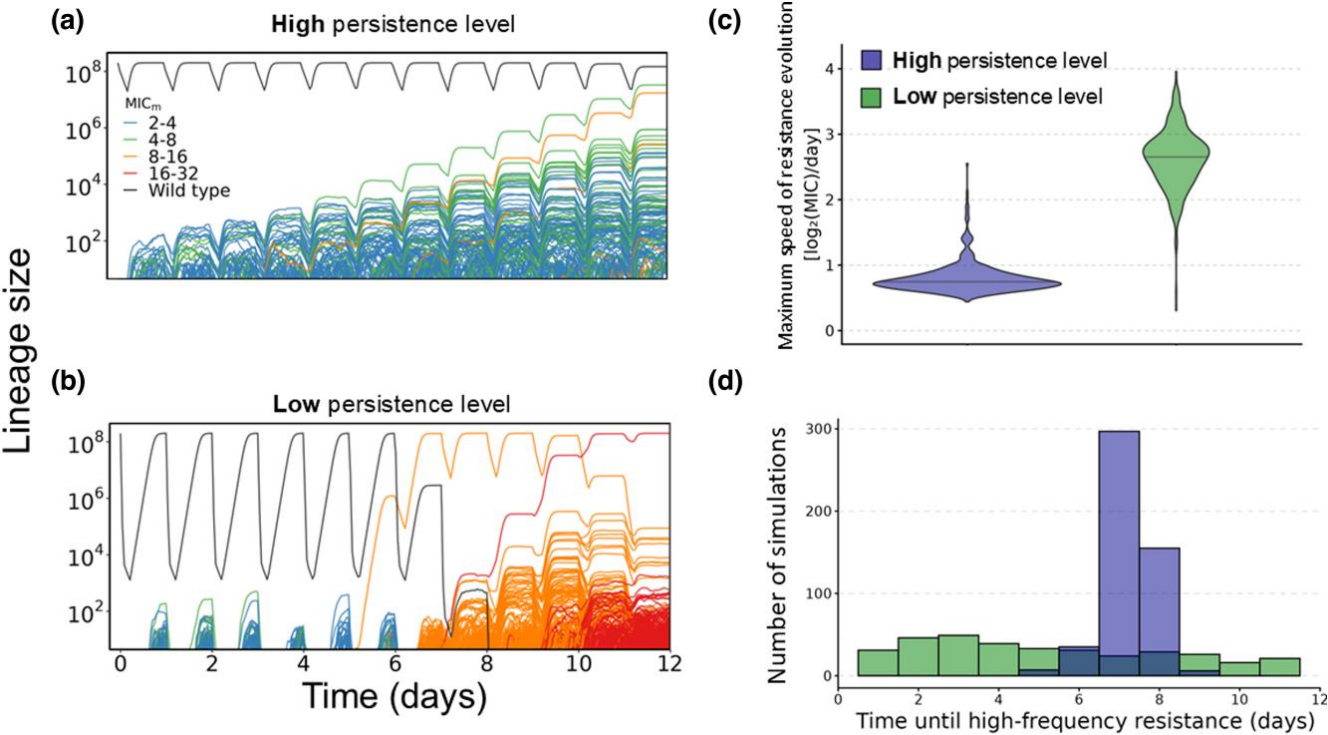
Persistence affects the speed and the predictability of the onset of antibiotic resistance evolution. The lineage sizes of the wild type (MIC = 2) and each resistant mutant lineage that arises in a single representative population with a) a high-persistence fraction (*α* = 0.8) and b) a low-persistence fraction (*α* = 5 × 10^−5^). Colors indicate the MIC of each lineage. Each day starts with a 5 h antibiotic treatment (12.5 μg/ml) followed by a regrowth period of 19 h (the antibiotic treatment window is visible in the decline of the wild-type population). c) Maximum rate of increase in MIC within any one 24 h window (log2(MIC)/day) across 500 high-persistence (blue) and low-persistence (green) simulations. Horizontal lines of the violin plot represent the mean. d) Histograms of the time at which any resistance mutation reaches 5% of the total frequency, for the same 500 high-persistence (blue) and low-persistence (green) simulations.

Figure 2a and b illustrates the evolution of resistance mutants in two representative populations with, respectively, high and low persistence levels, tracking the frequencies of both the wild-type cells (black) and the emerged mutant lineages over time (colors). The wild-type population under high persistence (Fig. 2a) exhibits relatively mild declines during antibiotic treatment, resulting in a large pool of survivors. Under these conditions, small-effect resistance mutations can persist, but their selective advantage is limited by strong competition from the abundant wild-type cells during the regrowth phase. This is further compounded by the fact that many such mutants often emerge within a short time span, intensifying clonal interference and slowing the speed of evolution. Under low persistence (Fig. 2b), most mutants that emerge between treatment cycles go extinct once the antibiotic is applied. However, when a rare, large-effect mutation occurs, it rapidly outcompetes the wild-type population, leading to its quick establishment. This is facilitated by minimal clonal interference from low-resistance lineages, as these tend to go extinct. As a result, across many simulations, these contrasting dynamics produce qualitatively distinct evolutionary outcomes depending on the persistence level. Resistance tends to increase much more quickly under low persistence than under high persistence (Fig. 2c), but the timing of resistance emergence is far less predictable (Fig. 2d).

### Persistence affects the magnitude of resistance evolution

**Figure 3:**
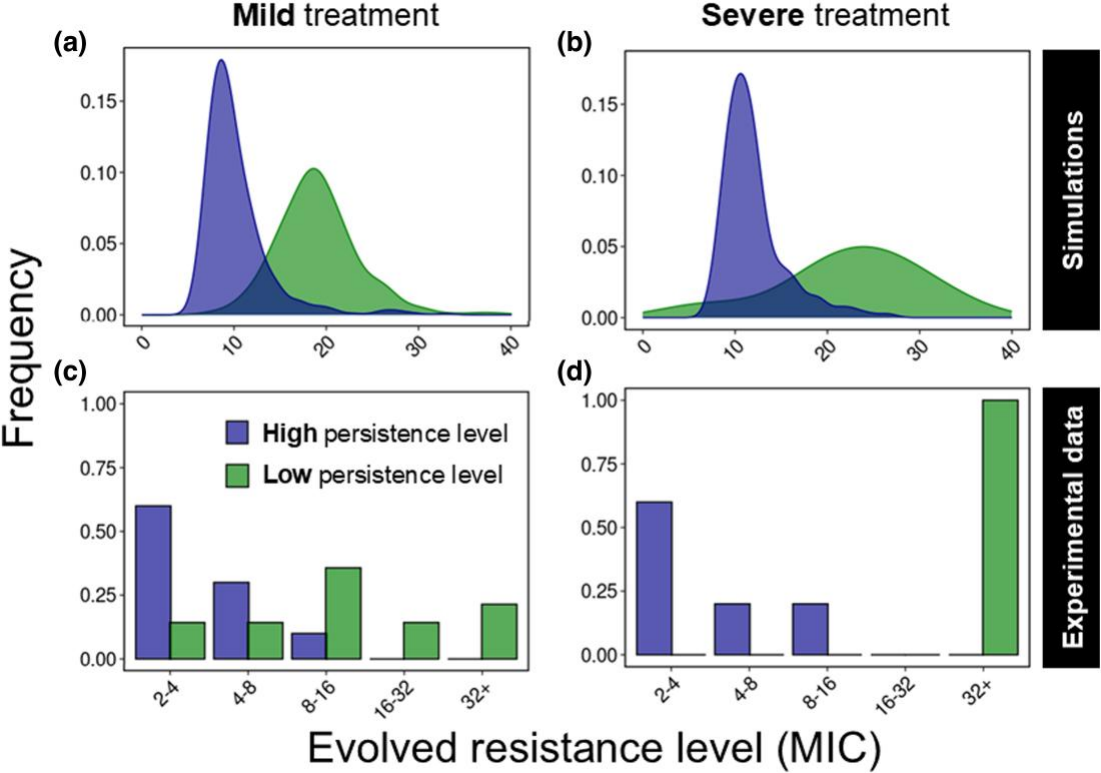
Persistence affects the magnitude of antibiotic resistance evolution. a, b) Distributions of the final MIC values after 12 d of treatment across 500 replicate simulations with a high-persistence fraction (0.8, blue) and a low-persistence fraction (5 × 10^−5^, green) under a) relatively mild antibiotic treatment (12.5 μg/ml) and b) more severe antibiotic treatment (25 μg/ml). c, d) MIC measurements of high-persistence (blue) and low-persistence (green) populations at the end of an evolutionary experiment under c) relatively mild antibiotic treatment (12.5 μg/ml amikacin) and d) more severe antibiotic treatment (25 μg/ml amikacin). For each combination of antibiotic concentration and persistence level, there are 24 replicate populations.

Our simulation model predicts that the contrasting dynamics under high and low persistence also generate distinct effects on the magnitude of resistance mutations. By the end of treatment, high-persistence populations tend to exhibit relatively modest resistance, whereas low-persistence populations reach high resistance levels (Fig. 3a and b, blue vs. green distributions). This qualitative difference in the distribution of resistance levels is consistent across treatments of different severity. To test these model predictions, we reanalyzed the data of an earlier evolutionary experiment (Windels et al. 2024) and sequenced the evolved populations. In this experiment, an *E. coli* strain was subjected to 12 consecutive 24 h cycles, each consisting of 5 h of antibiotic exposure followed by 19 h of regrowth, as in our simulations. This experiment was conducted in two distinct conditions that varied in nutrient concentration, resulting in considerably different environmentally induced persistence levels (with a low nutrient concentration resulting in higher persistence; see Figure S1). Hence, although this does not provide a direct manipulation of persistence level (which would require working with genetically different strains, leading to possible epistatic interactions with resistance mutations), we treat this difference in nutrient level as a proxy for persistence level in our empirical results.

After evolution, the populations were screened for their MIC to assess resistance levels. Consistent with our model predictions, high-persistence populations typically showed only modest or no increases in resistance (Fig. 3c and d, blue bars), whereas a considerable fraction of low-persistence populations attained substantially increased resistance (Fig. 3c and d, green bars). Moreover, higher antibiotic concentrations amplified this effect: All low-persistence populations that survived severe treatment evolved very high resistance levels (Fig. 3d, green bar). These results are qualitatively in line with our model predictions, although it is worth noting that the empirically evolved resistance levels may be due to epistatic effects in some cases, which were not included in our model.

### Persistence affects the number and type of mutations that establish

**Figure 4:**
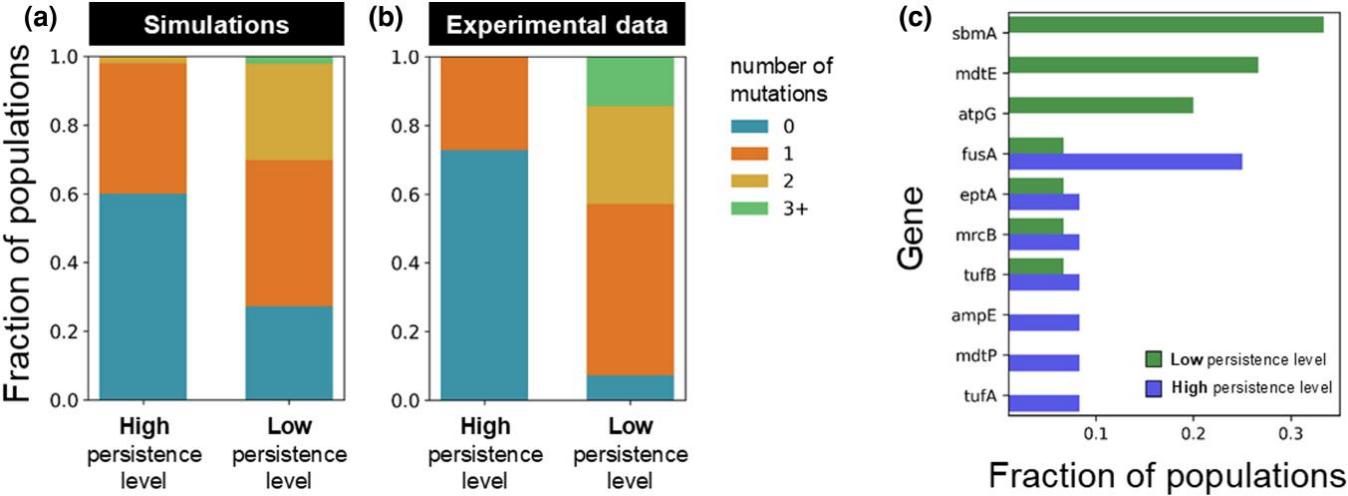
Persistence affects the number and type of mutations that arise. a) Number of mutations that reached high frequency (>80%) at the end of the simulation, across 500 replicate simulations per persistence level. b) Number of mutations that reached high frequency (>80%) at the end of the evolutionary experiment estimated with the sequencing data, across 27 replicate populations (see details in Table S1). For sensitivity analysis of chosen % threshold, see Figure S2. c) Fraction of replicate populations that harbored (at any frequency) the 10 most commonly mutated putative resistance genes pooled across both antibiotic treatments (12.5 and 25 μg/ml amikacin).

Next, we investigated how many individual mutations reached high frequencies (>80%) by the end of antibiotic treatment, again comparing our simulation results with the empirical data. Also in this case, the empirical data based on whole-genome sequencing of samples from the evolved populations and gene ontology (GO) analysis (see Materials and methods) are consistent with our model predictions. In both the model and the experiment, the vast majority of evolved high-persistence populations harbored no more than a single mutation that reached high frequency (Fig. 4a and b, leftmost bars). Although many mutations conferring low resistance may have been present in these populations, these mutations may have only reached low frequencies by the end of treatment due to the slow resistance evolution under high persistence (see Fig. 2a). In low-persistence populations, we instead observe relatively many mutations reaching high frequencies, with quite a few populations harboring two or more high-frequency mutations by the end of treatment (Fig. 4a and b, rightmost bars). These findings qualitatively agree with our model predictions and may be explained by similar mechanisms, ie the increased speed of resistance evolution under low persistence.

Interestingly, the experimental results show that the mutations in low- and high-persistence populations tend to occur in different genes. Figure 4c shows the fraction of replicate populations that had mutations in genes putatively associated with resistance (see Materials and methods for details). Although tentative, these results suggest that mutations in low-persistence populations tend to be in genes more commonly associated with antibiotic resistance, whereas this is less often the case for high-persistence populations. Specifically, low-persistence populations tended to exhibit more mutations in genes that have previously been characterized (Munck et al. 2014; Jahn et al. 2017) as impacting resistance (eg smbA, mdtE, and atpG), with fusA as the only clear exception. This pattern is consistent with resistance-associated mutations establishing more often in low-persistence populations. However, mutation occurrence alone cannot identify mutational effect sizes, as high MIC could arise from single large-effect mutations, combinations of smaller-effect mutations with nonadditive effects or condition-dependent pleiotropic effects. In addition, differences in nutrient concentration might have contributed to these differences.

### Persistence shapes a trade-off between survival and resistance evolution

**Figure 5:**
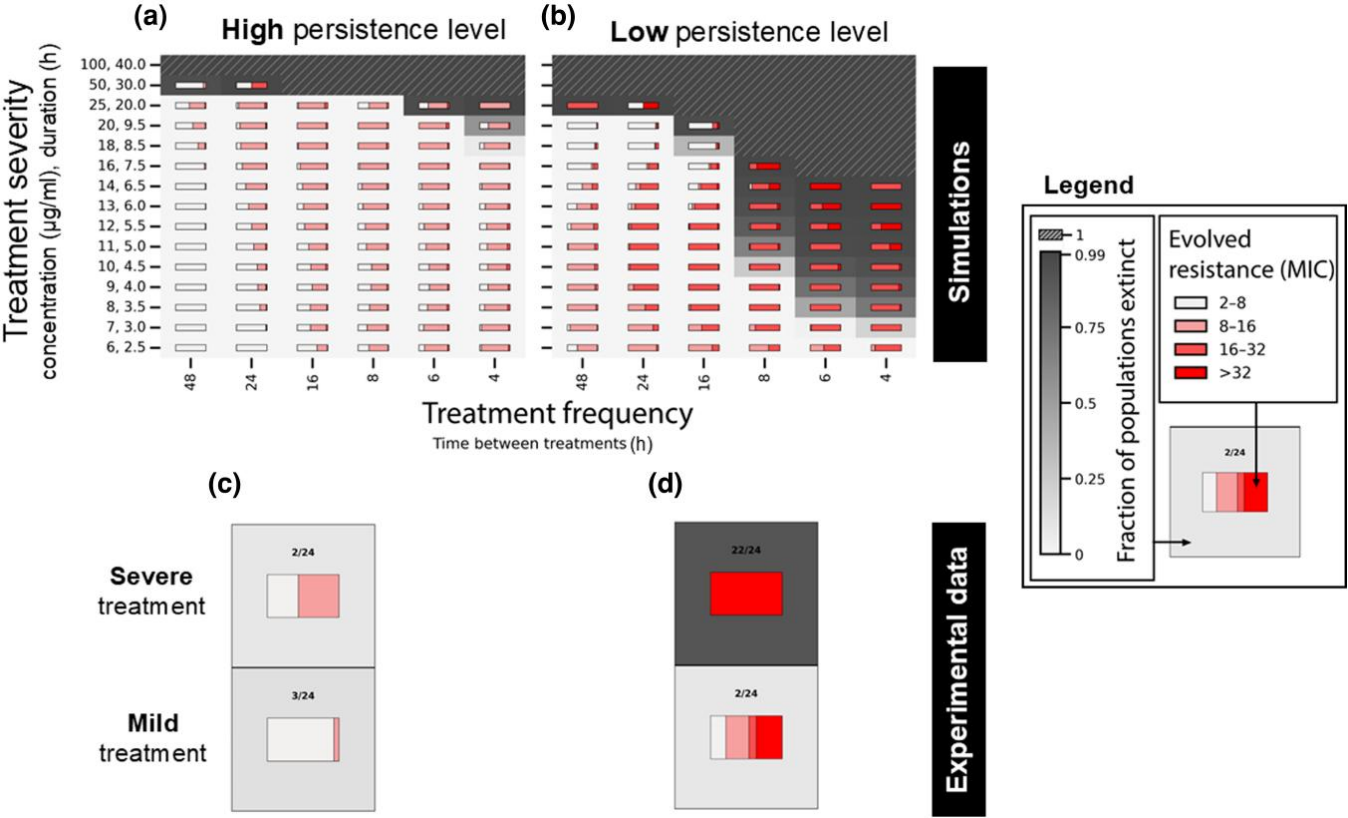
More severe and frequent antibiotic treatment risks high resistance evolution, especially in low-persistence populations. The graphs show both the fraction of extinct populations (shading) and the evolved resistance level (MIC; bars) at the end of antibiotic treatment, for populations with a, c) a high-persistence fraction and b, d) a low-persistence fraction. For each combination of treatment severity and frequency, the graphs show the fraction of a, b) 500 replicate simulations or c, d) 24 replicate experimental populations that went extinct (background color, with hatched black and white indicating the extinction of all populations), and a stacked bar showing the resistance level attained across all replicates (where each fraction corresponds to the fraction of replicate simulations that attained the associated resistance level). c, d) Experimental populations were exposed to mild (12.5 μg/ml amikacin) or severe antibiotic treatment (25 μg/ml amikacin), in both cases with 5 h of treatment alternated with 19 h of regrowth for 12 d. Above the MIC bar, the raw number of experimental replicate populations that went extinct (Extinct replicate populations/Total number of replicate populations).

Next, we investigated how the patterns we described above affect the evolution of antibiotic resistance across a wide range of antibiotic treatment regimens. We conducted an extensive simulation study where we systematically varied treatment severity (antibiotic concentration and duration of exposure) and frequency (length of the interval between treatments) for both high- and low-persistence fractions (see Fig. 5a and b, respectively), running 500 replicate simulations per condition. We kept track of the frequency of population extinction among these replicate simulations and of the evolved MIC level by the end of the simulated treatment (for the replicate populations that survived treatment). As above, we compare our results with the outcomes of the experiment (Fig. 5c and d).

Our simulations show that the evolution of high-persistence populations proceeds similarly across most treatment regimens (Fig. 5a). Although extremely severe treatment can eradicate even highly persistent populations, most regimens led to low extinction rates and relatively modest resistance evolution. In contrast, treatment severity and frequency have a strong impact on what happens in low-persistence populations, both regarding population survival and resistance evolution (Fig. 5b). Severe treatments are more likely to eradicate low-persistence populations, but they also risk the evolution of high levels of antibiotic resistance if these populations survive. Thus, the choice of antibiotic treatment regimen becomes far more consequential in populations with low-persistence levels. The experimental results are in qualitative agreement with our model outcomes: high-persistence populations generally survived but evolved only modest resistance, while low-persistence populations often went extinct under severe treatment, but those that survived frequently evolved high resistance levels (Fig. 5c and d). These results highlight an important principle: low persistence enhances resistance evolution only when antibiotic treatment is insufficiently severe to reliably cause extinction.

**Figure 6:**
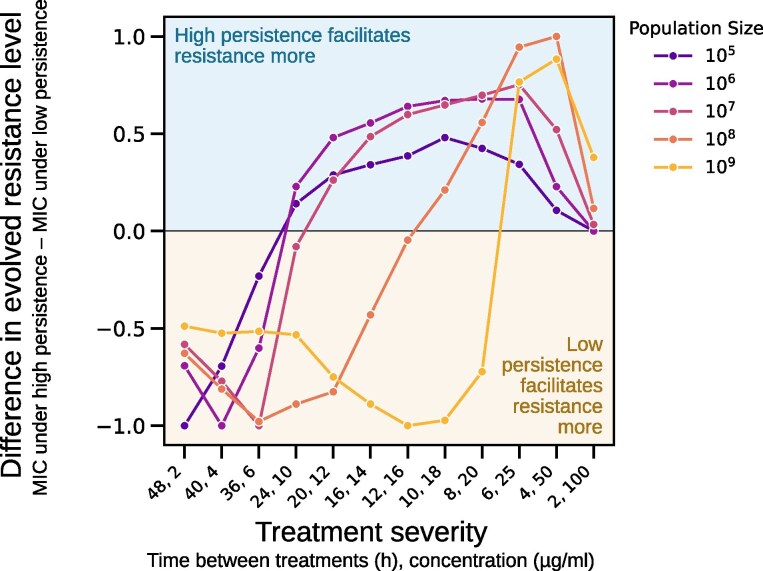
Persistence facilitates resistance evolution more in small populations than in large populations. The difference in attained average resistance level (MIC) between populations with a high- and a low-persistence fraction, depending on treatment severity and population size. The bottom half of the graph (in yellow) indicates the zone where higher average resistance is attained in low-persistence populations, while the top half (in blue) indicates the zone in which the opposite is the case. Each point shows the difference in average MIC attained in 500 replicate simulations for high persistence and 500 replicate simulations for low persistence.

Figure 5 shows that high resistance evolution is enhanced in low-persistence populations, but only if the antibiotic treatment is not severe enough to reliably drive the population extinct. A final consideration is that population size affects the range of treatment regimens for which this is the case. As it is well established in evolutionary theory, larger populations have an intrinsically greater capacity to survive drug exposure and generate new mutations, which provides relatively greater benefits to low-persistence populations. The ramifications of this are shown in Fig. 6. As before, low-persistence populations reach higher resistance levels than high-persistence populations when treatment is relatively mild, as they allow more mutational input driven by regrowth and a stronger selective advantage for resistant mutants, combined with a low risk of extinction. However, when we increase treatment severity, there is a clear tipping point beyond which high-persistence populations evolve higher resistance, as the extinction risk of low-persistence populations becomes too high. Population size has a clear effect on the location of this tipping point. In small populations, high persistence already facilitates resistance evolution more under relatively moderate antibiotic treatments, while in large populations, high persistence only becomes more facilitating if treatments are quite severe.

## Discussion

In this study, we conducted a systematic investigation of how persistence impacts the evolution of antibiotic resistance across treatment conditions. Our results partly align with previous findings (Levin-Reisman et al. 2017; Levin-Reisman et al. 2019; Windels et al. 2019) that reported a positive effect of persistence or complete tolerance on antibiotic resistance evolution. As first reported by Levin-Reisman et al. (2017), we also observe the effect that persistence can facilitate the establishment of partial resistance mutations from going extinct, allowing a gradual evolution of antibiotic resistance. However, here we include a continuous spectrum of resistance mutations and treatment severities and show that high persistence can also impede antibiotic resistance evolution. Specifically, even though individual resistance mutations tend to avoid extinction in high-persistence populations, the final evolved resistance level at the end of antibiotic treatment tends to be higher under low persistence. More generally, our model shows that persistence modulates a trade-off between the short-term survivability of the population and its long-term capacity for evolving high resistance levels. This trade-off can play out in different ways depending on the antibiotic treatment regimen and population size, which offers general insights for the design of treatment strategies.

By explicitly modeling how mechanisms affecting survival and growth shape resistance evolution across conditions, our model provides a formal basis in support of earlier arguments that biofilms may slow resistance evolution (Trubenová et al. 2022a, 2022b) and that biocidal drugs which cause much higher killing rates than biostatic drugs are more likely to lead to resistance evolution (Czuppon et al. 2023). Our results also agree with previous results that the distribution of mutational effects impact whether the evolutionary route to high resistance proceeds gradually or in large steps (Chevereau et al. 2015; Salverda et al. 2017) and with earlier work demonstrating that demographic bottlenecks and their associated effects on stochasticity can have complex consequences for the establishment probabilities and evolutionary trajectories of mutant lineages (Wahl and Gerrish 2001; Gamblin et al. 2023). More broadly, our results show that persistence has the potential to modify many of the variables that earlier studies have pointed out as the primary drivers of resistance evolution, including evolutionary history (Palmer et al. 2015; Santos-Lopez et al. 2019; Aggeli et al. 2021; Cisneros-Mayoral et al. 2022), mutation supply (Salverda et al. 2017; Ruelens and de Visser 2021; Ruelens et al. 2023), selection strength and allele competition (Toprak et al. 2011; Ogbunugafor and Eppstein 2016; Jahn et al. 2017).

By showing that persistence acts as a modifier of both selection strength and mutation supply, our model echoes recent observations that some traits (genetic or phenotypic) can increase or decrease the establishment chances of future mutations (Ferrare and Good 2024). This phenomenon has primarily been discussed in the context of the evolution of mutational robustness and evolvability (Riederer et al. 2022; Tawfeeq et al. 2024), but had (to our knowledge) not yet been considered in the context of antibiotic persistence and resistance evolution. This perspective is potentially powerful in this context, as it can help design antibiotic treatment strategies that minimize future resistance evolution. Our framework provides a basis for evaluating this risk, depending on aspects of the treatment regimen and the advancement stage of the infection (with more advanced infections typically exhibiting larger populations). For example, our model shows that the potential benefits of severe antipersistence treatment strategies (Defraine et al. 2018; Kaushik et al. 2022) might have to be weighed against the risk that very high resistance evolves if such treatment would still fail.

Although our model provides general insights, it also makes several simplifying assumptions that may limit its power to predict resistance evolution in the real world. Perhaps most importantly, our model assumes fixed persistence levels, whereas in nature, persistence can in some cases evolve quickly in response to antibiotic treatment (Van den Bergh et al. 2016; Carter et al. 2024). Our approach contrasts with earlier work (Levin-Reisman et al. 2017; Windels et al. 2019) in which persistence is assumed to evolve prior to resistance, based on the argument that persistence and population-wide tolerance evolve more readily because of their larger mutational target size (Girgis et al. 2012). However, recent research indicates that the complex causation of persistence makes its evolution complex and context-dependent: persistence can either evolve readily or not at all depending on the strain (Carter et al. 2024), the frequency of antibiotic application (Van den Bergh et al. 2016), metabolic dependencies of the antibiotic (Lobritz et al. 2015; Lopatkin et al. 2019; Zheng et al. 2022), and testing conditions (Conlon et al. 2016; Cabral et al. 2018; Geerts et al. 2022). Hence, through our assumption of fixed persistence levels, we provide baseline expectations that do not depend on specific assumptions about persistence evolution. This assumption also allows for a more direct comparison with our experimental results, where persistence levels are not genetically determined but rather environmentally triggered through differences in nutrient concentration. However, although environmentally triggered persistence is a well-established phenomenon (Balaban et al. 2019; Bollen et al. 2023), it also introduces potential confounds of persistence with the environmental trigger (in our case, nutrient level). This means that, even though our experimental results are in line with our theoretical predictions, they should be interpreted with considerable caution. The potential confounds with nutrient concentration may also be relevant with respect to population size—although we have not experimentally manipulated population size in this study, an experimental test of our model predictions pertaining to population size could lead to coupled physiological state through resource depletion, waste accumulation, pH, and respiratory activity and likely survivability which in turn may affect the evolutionary outcome.

Our model provides a general picture with relatively simple assumptions on the distribution of mutational effects on resistance and the pharmacodynamic effects of drug applications. We assume that resistance mutations additively increase MIC, which captures the sequential accumulation of multiple mutations but does not include explicit genotype-specific epistasis, where the effect of a mutation depends on the identity of previously acquired mutations. Such epistasis may be important: Combinations of individually modest mutations can generate high-level resistance (Ibacache-Quiroga et al. 2018; Wistrand-Yuen et al. 2018) and can interact synergistically with tolerance and resistance mutations (Levin-Reisman et al. 2019). Furthermore, these aspects will also vary depending on the specific species and antibiotic under consideration. In principle, it would be possible to use specific measurements to parameterize our model to obtain predictions for specific species-drug pairs, although such measurements can be notoriously difficult to obtain (eg with respect to mutation distributions). Regarding the conceptualization of persistence, our model specifically focuses on the treatment-survival aspects of the persister phenotype and does not consider possible differences in recovery dynamics, mutation rate, or DNA damage repair between persister and nonpersister cells. However, these differences may impact the evolutionary process, as antibiotic exposure and persister recovery can interact with stress responses, DNA repair, and error-prone polymerases, potentially altering the rate at which resistance mutations arise (Windels et al. 2019) and their selective advantage (Wahl and Zhu 2015). Finally, we make the simplest possible assumptions on the pharmacokinetics of the antibiotic, essentially mimicking an in vitro situation where the drug is either present at a fixed concentration or absent. This was a useful assumption for this study to be able to compare our model results with in vitro data, but our modeling framework can easily accommodate more complex pharmacokinetic assumptions to investigate in vivo scenarios.

Beyond opportunities to tailor our model to more specific combinations of species, antibiotic, and host, our modeling framework offers several potential avenues for further research. For example, as many infections occur in the form of biofilms, our framework could be extended to investigate how spatial structure modulates the persistence-resistance interplay (Trubenová et al. 2022a). This could shed light on how local variation in persistence levels across biofilm microenvironments may change the general implications for resistance evolution that we have shown. Another interesting opportunity entails the explicit modeling of multispecies infections, where the different species have different persistence levels, pharmacodynamic properties, and effective population sizes. This could elucidate how to best design treatment regimens when the likelihood of population extinction and the risk of antibiotic resistance evolution are species-dependent. Our framework could also be used to evaluate the impact of sequential, cyclical, and combination treatments (Aulin et al. 2021) on the balance between eradication success and the risk of resistance evolution, potentially providing possibilities to translate the insights of our work into clinical practice.

## Materials and methods

### Analytical model

#### Pharmacodynamics

We use a standard pharmacodynamic function after Regoes et al. (2004) that models the effect of the antibiotic concentration on the net bacterial growth rate (*ψ*), as follows:


(2) $$\psi (c)=\psi_{\min}\;\frac{1\;-\;{\left(\frac{c}{\mathrm{MIC}}\right)}^{h}}{\frac{\psi_{\min}}{\psi_{\max}}\;-\;{\left(\frac{c}{\mathrm{MIC}}\right)}^{h}}$$

where *C* represents the antibiotic concentration, MIC is the minimum inhibitory concentration (level of antibiotic resistance), *ψ*_ma*x*_ is the maximum net growth rate in the absence of the drug, *ψ*_min_ is the minimal net growth rate under high antibiotic concentration, and *h* determines the steepness of this function (the “Hill coefficient”). We assume that persistence cells have a less negative $\psi_{\min}$ compared to sensitive cells, which means they experience lower antibiotic-induced mortality (see Table 1 for all model parameters, including the specific values of *ψ*_min_ used for persistent and nonpersistent cells). This formulation is in line with the operational definition of persistence proposed by Balaban et al. (2019) in terms of the minimum duration for killing ($\mathrm{MD}K_{99}$), where the is the minimum duration required to kill 99% of the population. Specifically, the maximal killing rate ($\psi_{\min}$) of persisters used in our model is related to the MDK as follows: $\psi_{\min}=\;\ln(0.01)/\mathrm{MD}K_{99}$. Hence, the different aspects of the persistence phenotype are coarse-grained into the effective lower killing parameter $\psi_{\min}$ (or, equivalently, into the longer MDK).

**Table 1:** Overview of the parameters used in the evolutionary simulations. Unless explicitly mentioned otherwise, we assigned the mentioned values to these parameters in all simulations.

Parameter	Description	Value(s)
*b*	Birth rate	1.7
*d*	Death rate	1
*k*	Carrying capacity	$2\times 10^{8}$
*h*	Hill coefficient for antibiotic effect	2
$\psi_{\min(n)}$	Minimum growth rate (nonpersistent)	−6
$\psi_{\min(p)}$	Minimum growth rate (persistent)	−0.5
$\mathrm{MI}C_{\mathrm{wt}}$	Wild-type cell MIC (wild type) (μg/mL)	2
$\mathrm{MI}C_{m}$	Mutant MIC (μg/mL)	2−∞
*C*	Antibiotic concentration (μg/mL)	2-100
*α*	Persistence level	$10^{-5}-1$
$\mu_{r}$	Per-birth rate of resistance mutation	$10^{-6}$
DFE	Log-normal for MIC mutations	Mean = 0, *σ* = 0.07
AB cycle	5 h treatment + 19 h regrowth	Repeated up to 12 d
$N_{\mathrm{sim}\;}$	Independent simulation runs per condition	500

### Calculating survival under treatment

The proportion of a given (wild-type or mutant) lineage that survives antibiotic treatment $S_{i}$ depends on its MIC, the persistence fraction *α*, the antibiotic concentration *C*, and the duration of exposure *τ* (see also Levin-Reisman et al. 2017, 2019), as follows:


(3) $$S_{i}=(1-\alpha )e^{\psi_{n}(c,\mathrm{MIC})\tau }+\alpha e^{\psi_{p}(c,\mathrm{MIC})\tau }$$

where *ψ*_n_ and *ψ*_p_ are pharmacodynamic functions describing the net growth rates of the nonpersistence and persistence subpopulations, respectively, which differ only in their values of *ψ*_min_ (see above). In the model, *α* denotes the fraction of cells entering the protected survival state during antibiotic exposure. This spans a continuum from rare persistence to *α* = 1, representing the limiting case of population-wide tolerance-like slow killing. See SI Appendix for a full derivation of the analytical model.

## Stochastic simulation model

We implement a version of the Gillespie algorithm that models a birth-death process and keeps track of the sizes of the different lineages that are present in the population (the wild type and all mutants). Each simulation is initialized with a single monomorphic population of size 2 × 10^8^ (which is also the carrying capacity *K*) and an initial MIC of 2. In the absence of the antibiotic, a number of births $B_{i}$ is drawn for each lineage in every time step (accounting for density regulation), according to $B_{i}∼\mathrm{Poisson}(b(1-N/K)N_{i})$, where *b* is the birth rate, *N* is the total population size, and $N_{i}$ is the lineage size (see Table 1 for a complete overview of model parameters). Similarly, a number of deaths $D_{i}$ is drawn according to $D_{i}∼\mathrm{Poisson}(dN_{i})$, where *d* is the death rate. At the start of every antibiotic pulse, a fraction of each subpopulation undergoes a stochastic switch to the persister phenotype. If the size of a subpopulation immediately before the pulse is *N_i_*, the number of cells that enter persistence is drawn from $Np_{i}∼\mathrm{Poisson}(N,\;\alpha )$, with *α* the global persistence level (*α* = 0.8 for high-persistence level and *α* = 5 × 10^−5^ for low-persistence level, unless stated otherwise). The net growth rate of each lineage is determined by *ψ* according to (2) (where *ψ*_min_ is equal to *ψ*_min(n)_ for nonpersistence cells and *ψ*_min(p)_ for persistent cells; see Table 1), and this is again used to parameterize a Poisson distribution to draw the number of births or deaths during antibiotic treatment (depending on the MIC level), as above. We assume that deaths not directly caused by the antibiotic still occur during antibiotic treatment, so we add the deaths caused by the regular death rate and the deaths from the antibiotic treatment to obtain total deaths. At any birth event, a mutation occurs with probability *μ*, in which case a number drawn from a log-normal distribution with a log-scale mean 0 and standard deviation *σ* is added to the parental MIC value to determine the MIC value of the new mutant lineage (this distribution was chosen as a generalization of recent empirical evidence^18^—see Figure S3 for alternative parametrizations).

At the end of each simulation, we recorded the frequency distribution of the surviving subpopulations and their MIC values. For each parameter combination, we performed 500 independent replicate simulations and recorded the fraction of simulations that resulted in complete extinction of all lineages as well as the average MIC by the end of the simulation of all nonextinct simulations. All simulations were implemented in *Python* using the tau-leaping Gillespie algorithm for stochastic simulation and parallelized with *mpi4p*.

### Evolutionary experiment

The evolved strains originate from an independent experimental evolution study (Windels et al. 2024) using the *E. coli* SX43 strain, a derivative of BW25993 (from Van den Bergh et al. 2016). Cultures were grown at 37 °C in Mueller–Hinton broth (500 μl MHB) with orbital shaking (1.5 mm shaking orbit; 1,200 rpm). Experimental evolution was performed in 96-well plates with 24 parallel replicates per treatment condition over 12 d. Daily cycles included a 5 h treatment with the aminoglycoside amikacin, followed by washing (using 10 mM MgSO_4_ to remove the antibiotic) and a regrowth phase in fresh MHB. Prior to treatment, cultures were diluted to reach two nutrient conditions (25% MHB and 80% MHB), and cell density was monitored by spot plating on LB agar. These conditions resulted in different survival at the beginning of the evolutionary experiment that we interpret as the realized, environmentally induced persistence level (see Figure S1 for details). Note that nutrient conditions were only varied during the antibiotic treatment phase—they were the same between the two conditions during the regrowth period. The MIC was determined via a standard broth microdilution method using a series of twofold amikacin dilutions. Optical density readings after 24 h of incubation were used to assess growth and assign MIC values.

### Whole-genome sequencing and resistance mapping

#### Sequencing

Genomic DNA was extracted from the evolved populations at the conclusion of the experiment. Making use of the DNeasy blood and tissue kit (Qiagen), we assessed purity (Nanodrop), quantity (Qubit), and stability (gel electrophoresis). Next, indexed NGS sequencing libraries were prepared using an adapted, multiplex protocol following the enzymatic xGen EZ DNA Library Prep Kit (IDT). Following bead purification and quality control (eg insert size ± 300 bp, no adapter/index dimers, and concentration), libraries were pooled and converted to Element-compatible libraries using the Adept kit. Converted pools were then sequenced on the AVITI system (Element, by the Nucleomics Core VIB—Leuven) to generate high-quality paired-end sequence data. Demultiplexed reads underwent quality control (FastQC and MultiQC), trimming (Trimmomatic), and filtering and were then aligned to the appropriate *E. coli* reference genome. Variants and their respective frequencies were called using the breseq computational pipeline (Deatherage and Barrick 2014) for finding mutations relative to the reference sequence (K12 MG1655 NC 913000.3). Raw fastq files were deposited on the SRA database with accession number PRJNA498891.

#### Post-processing and frequency estimation

Following variant calling and annotation of whole-population sequencing data, we estimated the frequency of high-frequency mutations across replicate evolved populations. Within each replicate, mutations achieving a frequency greater than 80% were considered “high-frequency” (for sensitivity analysis, see Figure S2). For each replicate population, the number of unique high-frequency mutation events was determined. Finally, the fraction of replicates falling into each mutation count category (0, 1, 2, or 3+ high-frequency mutations) was calculated for each condition, providing a quantitative basis for comparative analyses.

#### Functional analysis

To infer the functional significance of the observed mutations, we relied on the dataset from Jahn et al. (2017), and we integrated via GO annotations with our variant data. Using a current GO analysis database, all GO annotations (Biological Process, Molecular Function, and Cellular Component) for *E. coli* K-12 MG1655 were extracted from the Gene Ontology Consortium repository (Gene Ontology Consortium, 2025, https://github.com/geneontology/go-site). The ontology structure (go.obo) corresponded to the 2025-02-06 release. Annotation parsing and term propagation were carried out with GOATOOLS v1.4.5 under Python 3.11 (Klopfenstein et al. 2018). A critical filtering step was then applied to identify resistance-related functions. Specifically, GO terms were flagged as relevant if their cellular function contained the key “antibiotic” or “resistance.”

## Supplementary Material

msag180_Supplementary_Data

## Data Availability

The data and code underlying this article are available at https://doi.org/10.6084/m9.figshare.31389142. The raw sequencing data can be accessed on the SRA database with accession number PRJNA498891.
