# Supplementary material for: Bacterial Persistence Modulates the Speed, Magnitude, and Onset of Antibiotic Resistance Evolution
Source: Mol Biol Evol. 2026 Jul 22;43(8):msag180. doi: 10.1093/molbev/msag180 (PMC13475807; doi:10.1093/molbev/msag180)
Supplement: Supplementary file 1 [file msag180_supplementary_data.pdf]

Supplementary Information for

Bacterial persistence modulates the speed, predictability, and magnitude of antibiotic resistance evolution

Giorgio Boccarella, Philip Ruelens, Ernesto Berríos-Caro, Bram Van den Bergh, Lloyd Cool, Jan Michiels, Pieter van den Berg

Corresponding author: Giorgio Boccarella

Email: giorgio.boccarella@kuleuven.be, piet.vandenberg@kuleuven.be

This PDF file includes:

Supplementary text

Figures S1 to S3

Table S1

Supplementary Information Text

Full derivation of analytical model

We aim to calculate the probability that at least one resistance mutant arises during regrowth and ultimately survives a subsequent antibiotic treatment. We term this probability as emergence probability. The derivation of this probability combines three key elements: mutation occurrence, stochastic mutant growth, and survival after treatment.

1) Mutation emergence during regrowth

Let us assume a wild-type population that starts at size N_a at time $t = 0$ and grows for a duration T during the regrowth period. We consider growth in continuous time. The population grows exponentially and deterministically following

$$n_{\mathrm{wt}}(t) = N_a e^{r_{\mathrm{wt}} t}$$

where $r_{\mathrm{wt}} = b_{\mathrm{wt}}(1 - \mu_r(r)) - d_{\mathrm{wt}}$ is the intrinsic growth rate of the wild-type population, with b_{wt} and d_{wt} as the birth and death rates, and $\mu_r(r)$ the per-division mutation probability. We consider T as the time required for the population to regrow from N_a back to its original size N_b, i.e.,

$$T = (1/r_{\mathrm{wt}}) \log{(N_b/N_a)}$$

Mutants with resistance level r arise during wild-type divisions with probability $\mu_r(r)$ and grow stochastically following a birth-death process with birth rate b_M and death rate d_M. We define their resistance level as $r = \mathrm{MIC}_m/c$, where MIC_m is the MIC of the mutant population and c the antibiotic concentration during the antibiotic treatments. We write the mutation probability as $\mu_r(r) = \mu_{\text{total}}\, f_{\mathrm{LN}}(r)$, where $f_{\mathrm{LN}}(r)$ is assumed to follow a log-normal distribution, i.e.,

$$f_{\mathrm{LN}}(r) = \frac{1}{r\sigma\sqrt{2\pi}} \exp\left(-\frac{(\log\, r)^2}{2\sigma^2}\right)$$

with $\sigma > 0$ the logarithm of scale.

The total rate at which mutants with resistance level r arise at time t is given by

$$\lambda(r, t) = b_{\mathrm{wt}} \mu_r(r) n_{\mathrm{wt}}(t)$$

This defines a non-homogeneous Poisson process with time-dependent rate $\lambda(r,t)$ over the interval $[0,T]$. The expected number of mutants arising in a small time-interval $[t, t+\Delta t]$ is

$$\Lambda(r,t) = \lambda(r,t)\Delta t$$

2) Mutant establishment

Each mutant that appears during regrowth faces the possibility of extinction due to stochastic birth and death events. The probability that a mutant arising at time t establishes a lineage that survives by time T is

$$p_{\text{est,bdd}}(r,t) = 1 - p_{\text{ext,bd}}(t,T),$$

where $p_{\text{ext , bd}}\ (t,T)$ is the extinction probability of a birth-death process given by (see Bailey(Anon))

$$p_{ext,bd}(t,T) = 1 - \frac{r_M}{b_M - d_M e^{-r_M(T-t)}},$$

with $r_M = b_M - d_M$.

If a mutant survives regrowth, it must still endure the subsequent antibiotic treatment. We denote its probability of surviving treatment by $S_m(r)$. Assuming that survival after establishment and the treatment act independently, the total probability that a mutant appearing at time f persists through both phases is

$$q(r,t) = p_{ext,bd}(t,T) \cdot S_m(r).$$

3) Survival probability of mutants

Now consider a small time interval $[t, t+\Delta t]$, during which a number k of mutants may arise. The probability that none of these k mutants survives both regrowth and treatment is:

$$(1 - q(r,t))^k$$

To compute the overall extinction probability of the mutants (and their lineage) arising in this interval, we calculate the contributions from all the possible number of mutations

$$\mathbf{P}_{\text{ext},t} = \sum_{k=0}^{\infty} \mathbf{P}(k)(1 - q(r,t))^k$$

where $\mathbf{P}(k)$ is the probability that k mutants have appeared. Since $k \sim \mathrm{Poisson}(\Lambda)$, (i.e., $\mathbf{P}(k) = \Lambda^k e^{-\Lambda}/k!$), we find

$$\mathbf{P}_{ext,t} = e^{-\Lambda(r,t)\varphi(r,t)}$$

This expression arises from the Poisson thinning property (see Chiu et al. (Chiu et al. 2013)) in a Poisson process with rate Λ, if each event independently "succeeds" with probability q, then the number of successful events is Poisson-distributed with mean Λq. Hence, the probability of no successful events (i.e., no mutants surviving both stages) is $\exp(-\Lambda q)$.

To calculate the probability that no mutant arising over the entire regrowth window $[0, T]$ survives, we multiply the extinction probabilities across all the intervals. Let us divide $[0, T]$ into N subintervals $[t_n, t_n + \Delta t]$, where $t_n = (n-1)\Delta t$ for $n \in \{1,2, \dots, N\}$. We obtain

$$P_{\mathrm{ext}}(r) = \prod_{n=1}^{N} P_{\mathrm{ext},t_\infty} = \prod_{n=1}^{N} e^{-A(r,t_n)\gamma(x,t_\infty)} = e^{-\sum_{\mathrm{n}=1}^{N} \lambda(r,t_\mathrm{n})q(s,t_\mathrm{n})\Delta t}.$$

Taking the limit $N \to \infty$ and $\Delta t \to 0$, so the sums turn into integrals, we find

$$\mathrm{P}_{\mathrm{ext}}(r) = \exp\left[-\int_0^T \lambda(r,t)q(r,t)\mathrm{d}t\right].$$

Finally, the probability that at least one mutant survives - our desired quantity - is the complement of the extinction probability, i.e., the emergence probability

$$\mathbf{P}_{e,r} = 1 - \mathbf{P}_{\mathrm{ext}}(r) = 1 - \exp\left[-\int_0^T b_{\mathrm{wt}}\,\mu_r(r)n_{\mathrm{wt}}(t)p_{\mathrm{est,\,bd}}(t,T)S_m(r)\mathrm{d}t\right]$$

This integral formulation highlights how the emergence probability depends on both when a mutant arises and how likely it is to survive. Mutants that appear earlier during regrowth have more time to expand and are more likely to survive ($p_{\mathrm{est,\,bd}}(t,T)$ is larger), while those that appear late have less time to establish before treatment is applied.

Solving the integral over time, we find

$$\mathbf{P}_{e,r}(r) = 1 - \exp\left[-b_{\mathrm{wt}}\,\mu_r(r)\phi(T)S_m(r)\right]$$

where

$$\phi(T) = \frac{N_a r_M}{r_{\text{wt}} b_M}\left[e^{r_{\text{wt}}T}\ {}_2F_1\left(1, \frac{r_{\text{wt}}}{r_M}; \frac{r_M + r_{\text{wt}}}{r_M}; \frac{d_M}{b_M}\right) - \ {}_2F_1\left(1, \frac{r_{\text{wt}}}{r_M}; \frac{r_M + r_{\text{wt}}}{r_M}; \frac{d_M e^{-r_M T}}{b_M}\right)\right]$$

where ${}_2F_1$ is the Gaussian hypergeometric function (also known as the ordinary hypergeometric function). This result is similar to the probability of single resistance calculated in Berríos-Caro et al.(Berríos-Caro et al. 2021)

The term $S_m(r)$ is defined as

$$S_m(r) = (1-\alpha)S_{\text{sens}}\,(c, \tau, \text{MIC}_m) + \alpha S_{\text{tol}}(c, \tau, \text{MIC}_m)$$

If $\text{MIC}_m/c \gg 1$, then $S_m(r) \approx 1$ for every value of α (see Figure 1 of the main text). This means we expect $\mathbf{P}_{e,r}$ to not vary much as changing α when $\text{MIC}_m/c \gg 1$, implying that resistance is strong enough that even non-persistence cells survive.

4) Selection coefficient

Interestingly we can also define the selection coefficient s of the mutant compared to the wild-type during antibiotic treatment of duration τ as

$$s(r) = \frac{1}{\tau}\log\left(\frac{S_m(r)}{S_{\text{tot}}}\right)$$

where S_{tot} is the emergence probability of the total wild-type population, considering tolerant and non-tolerant cells. From this equation, we can express $S_m(r)$ as function of s as

$$S_m(r) = S_{\text{tot}}\, e^{\tau s(r)}$$

Inserting this in Eq. (14), we obtain

$$\mathbf{P}_{e,r}\,(r) = 1 - \mathbf{P}_{\text{ext}}\,(r) = 1 - \exp\left[-\int_0^T b_{\text{wt}}\,\mu_r(r) n_{\text{wt}}\,(t) p_{\text{est , bd}}\,(t, T) S_{\text{tot}}\, e^{\tau s(r)} \text{d}t\right]$$

From here we can deduce that

- Even small positive selection coefficients can lead to high values of the emergence probability due to the exponential boost from $e^{\tau s(r)}$.

- Mutants with higher r (i.e., higher MIC) tend to have higher $s(r)$, but may be rarer because of $\mu_r(r)$ that decays for large r, which may lead to a lower emergence probability.

All in all, low selection coefficient does not necessarily mean low emergence probability. In tolerant populations (high persistence fractions), resistance may survive even without strong selection, simply due to population dynamics and mutant input rate.

Figures

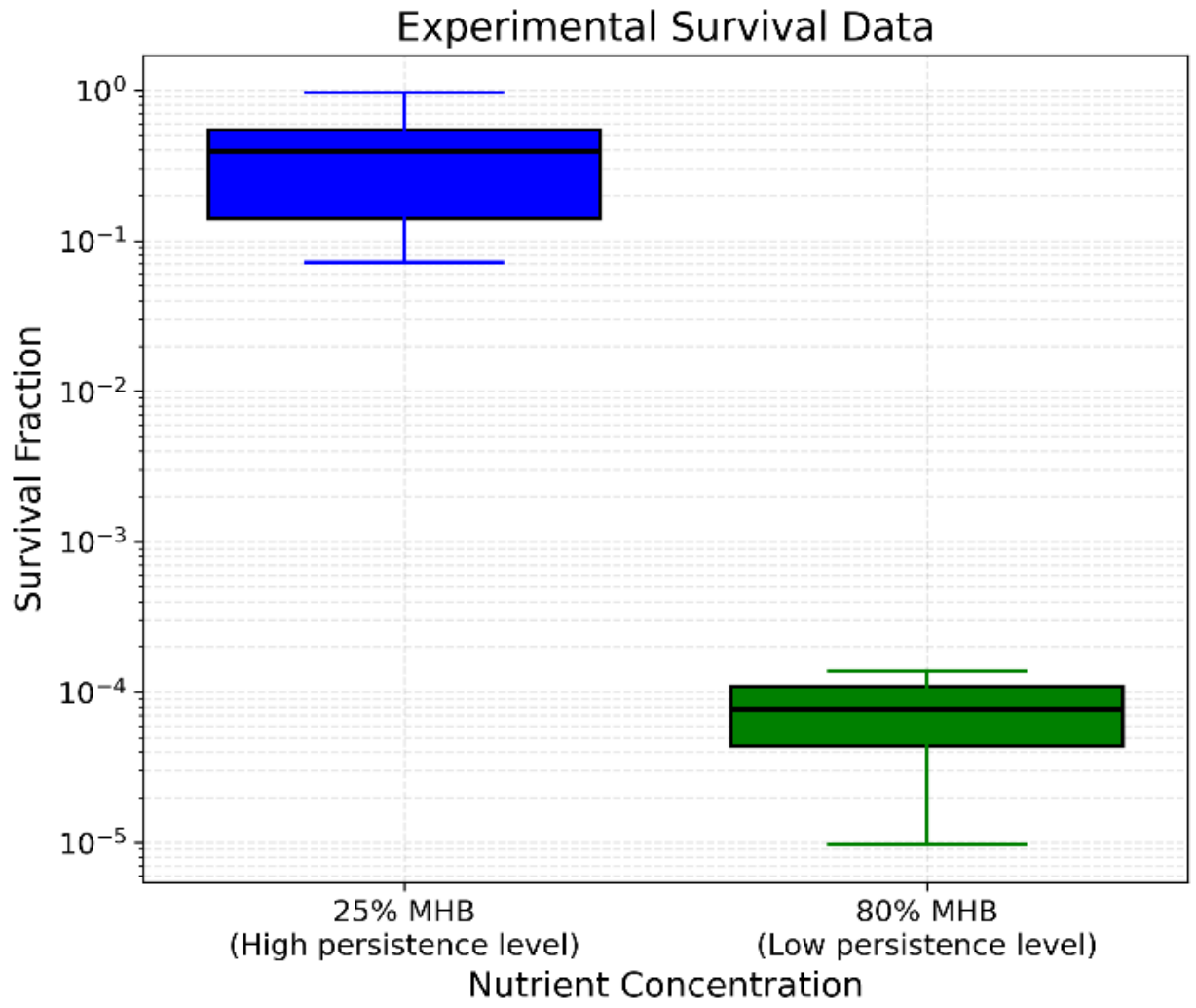

Figure S1. Survival of *E. coli* populations during a single bout of amikacin exposure is governed by nutrient level. Frequencies of surviving cells across 24 experimental replicates for each nutrient condition after a single 5-hour exposure to amikacin, measured on day one or two of the evolutionary experiment, in low (25 % MHB, blue) and high (80 % MHB, green) nutrient medium. Survival in low-nutrient medium is several orders of magnitude higher, consistent with a much higher induced persistence fraction.

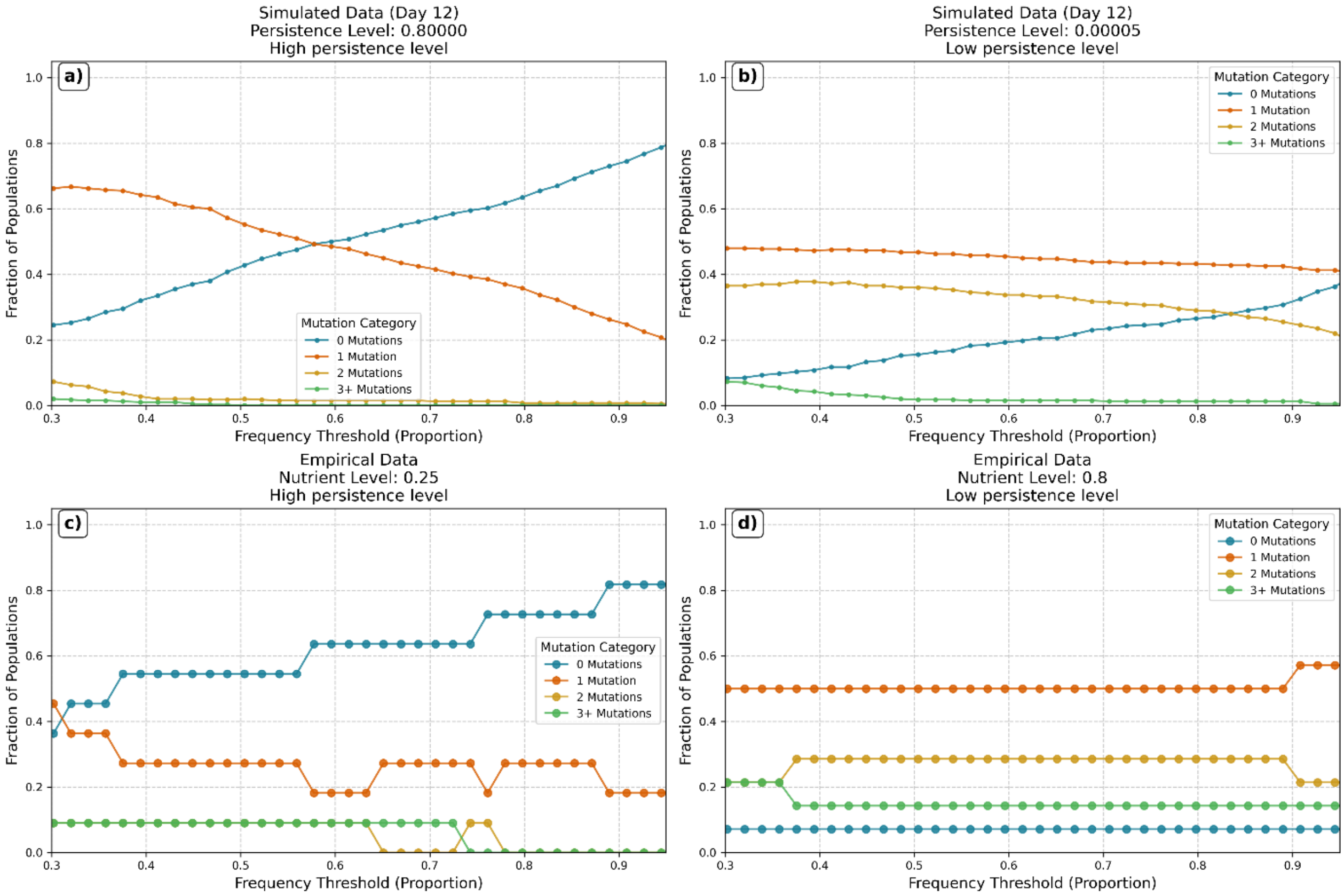

Figure S2. Frequency data of populations by mutation count for simulations and experimental sequencing data. The fraction of simulated populations that meet or exceed a given within-population frequency threshold is plotted for four mutation category: 0 mutations (blue), 1 mutation (orange), 2 mutations (gold), and ≥3 mutations (green). For high and low persistence level in simulations (a-b). (c-d) Show the fraction of replicate populations evolved in high and low persistence condition in which a certain number of mutation meet or exceed a given frequency threshold.

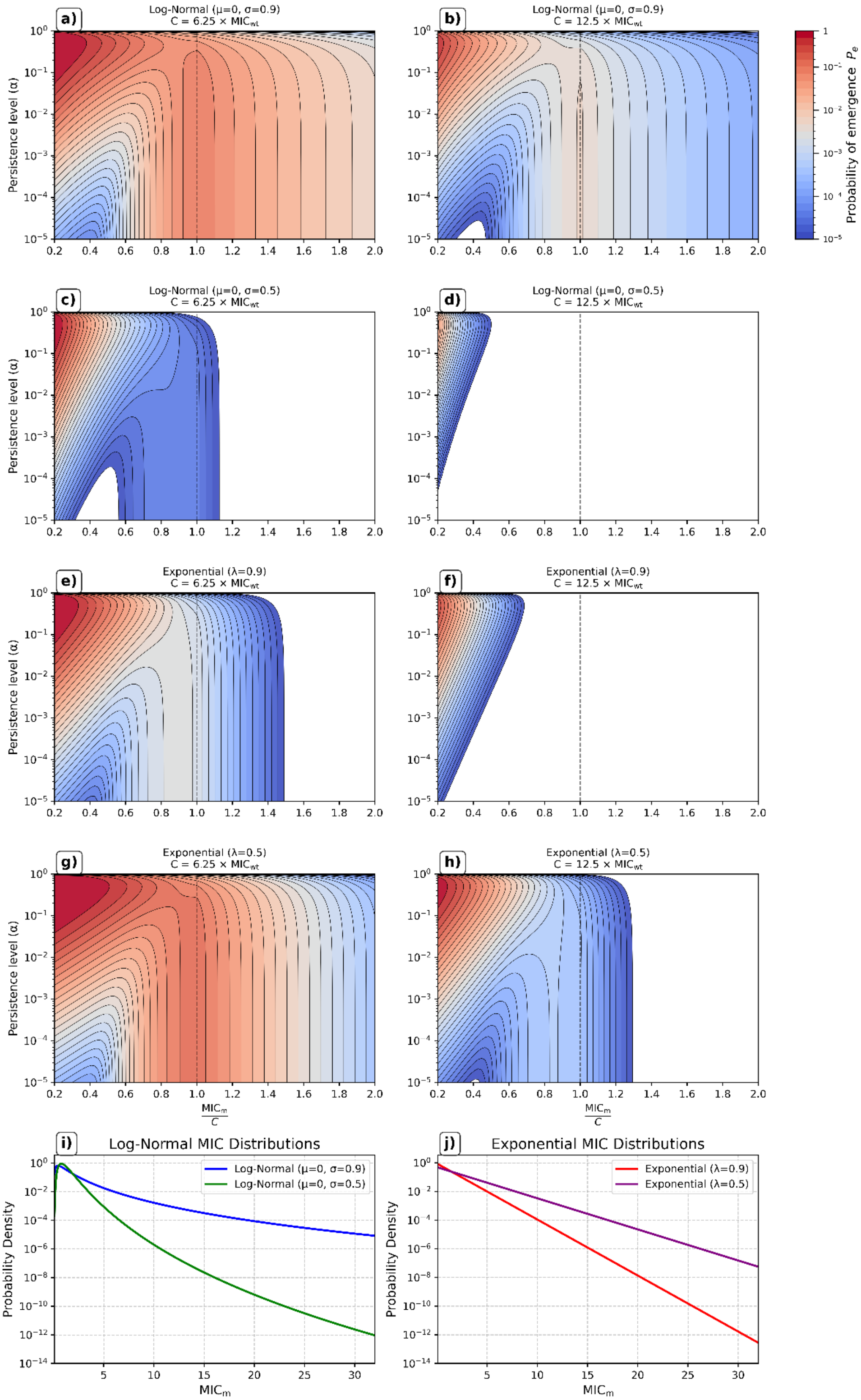

a)
Log-Normal (μ=0, σ=0.9)
C = 6.25 × MIC_wt
b)
Log-Normal (μ=0, σ=0.9)
C = 12.5 × MIC_wt
c)
Log-Normal (μ=0, σ=0.5)
C = 6.25 × MIC_wt
d)
Log-Normal (μ=0, σ=0.5)
C = 12.5 × MIC_wt
e)
Exponential (λ=0.9)
C = 6.25 × MIC_wt
f)
Exponential (λ=0.9)
C = 12.5 × MIC_wt
g)
Exponential (λ=0.5)
C = 6.25 × MIC_wt
h)
Exponential (λ=0.5)
C = 12.5 × MIC_wt
Persistence level (α)
MIC_m / C
Probability of emergence P_e
i)
Log-Normal MIC Distributions
Log-Normal (μ=0, σ=0.9)
Log-Normal (μ=0, σ=0.5)
j)
Exponential MIC Distributions
Exponential (λ=0.9)
Exponential (λ=0.5)
Probability Density
MIC_m

Figure S3. The emergence probability of resistance mutations, depending on persistence level and resistance index with different DFE parametrization. Contour plots display the emergence probability across varying persistence fractions (vertical axis) and resistance mutant $\mathbf{MIC}$ relative to the antibiotic concentration (horizontal axis). Warmer colors (reds and oranges) indicate higher establishment probabilities, while cooler colors (blues) represent lower probabilities. (a-d)implement a log-normal distribution of resistance effects, while (e-h) use an exponential distribution; within each pair the first row/column shows the broader ($\sigma = 0.9$ or $\lambda = 0.5$) distribution and the second the narrower ($\sigma = 0.5$ or $\lambda = 0.9$).

Tables

Table S1: Count of sequenced population for each persistence level and concentration

Persistence level	Antibiotic (μg/mL)	Count
High	$12.5(6.25 \times MIC_{wt})$	10
High	$25.0(12.5 \times MIC_{wt})$	2
Low	$12.5(6.25 \times MIC_{wt})$	13
Low	$25.0(12.5 \times MIC_{wt})$	2

SI References

Bailey NTJ. 1990. The elements of stochastic processes with applications to the natural sciences. New York, NY, USA: John Wiley & Sons.

Berríos-Caro E, Gifford DR, Galla T,. 2021. Competition delays multi-drug resistance evolution during combination therapy. J Theor Biol 509:110524.

Chiu SN, Stoyan D, Kendall WS, Mecke J. 2013. Stochastic Geometry and Its Applications. New York, NY, USA: John Wiley & Sons.
